# Supplementary figures and images for: Curated mitochondrial genome reference database of state key protected wild mammal in China
Source: PLoS One. 2025 Oct 23;20(10):e0335243. doi: 10.1371/journal.pone.0335243 (PMC12548896; doi:10.1371/journal.pone.0335243)

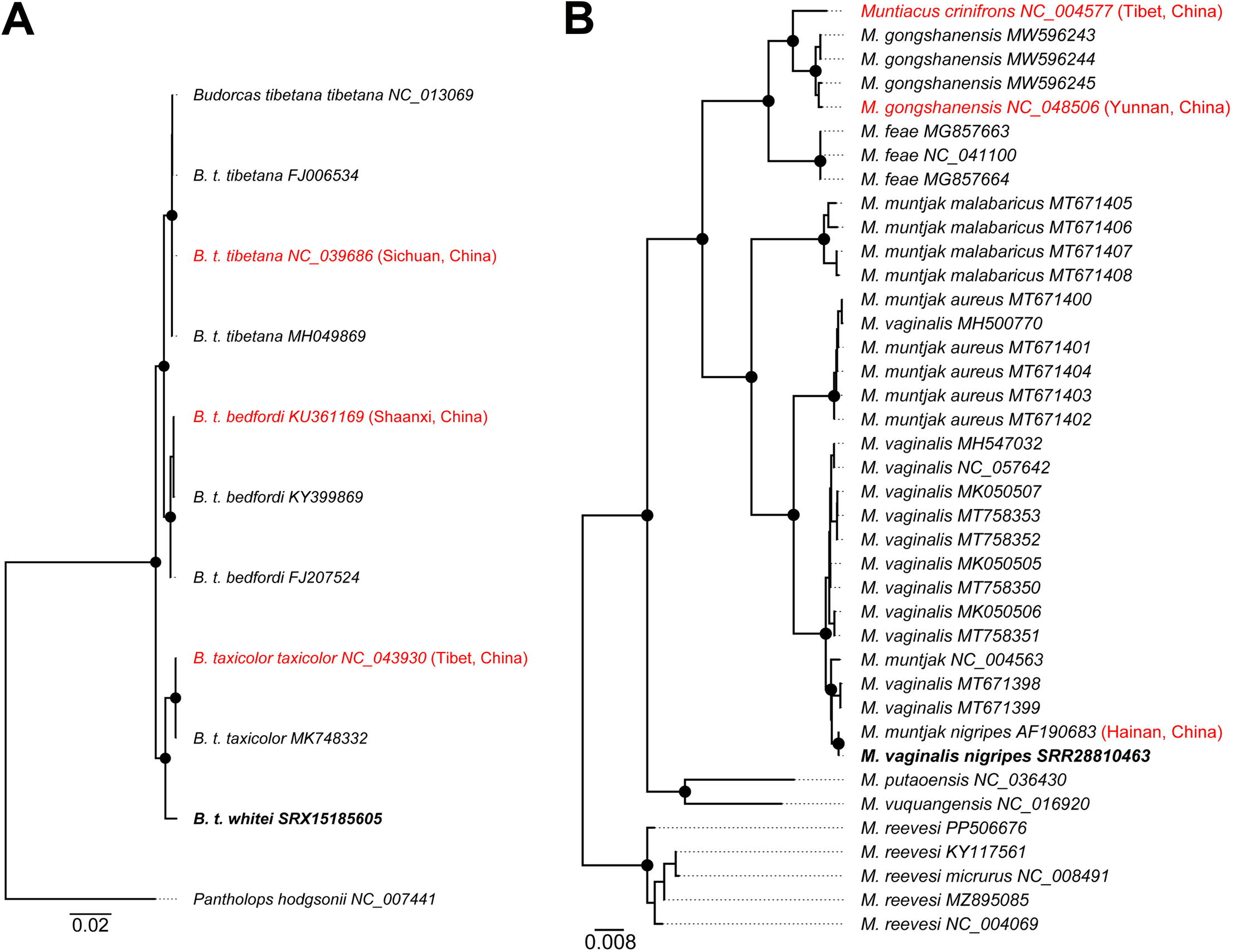

Supplement: S1 Fig — Nodes with BS ≥ 95 are indicated by black dots, while those with BS below 95 are shown as numbers. Bold names are newly obtained sequences in this study. Red names are designated curated mitochondrial genome sequence for species based on type locality and phylogenetic relationships. Sequences from GenBank used in this study are named with their accession numbers. (TIF) [file pone.0335243.s001.tif]
