# Supplementary material for: Curated mitochondrial genome reference database of state key protected wild mammal in China
Source: PLoS One. 2025 Oct 23;20(10):e0335243. doi: 10.1371/journal.pone.0335243 (PMC12548896; doi:10.1371/journal.pone.0335243)
Supplement: Attachment — Submitted filename: Response to Reviewers.docx [file pone.0335243.s004.docx]

Review Comments to the Author

Please use the space provided to explain your answers to the questions above. You may also include additional comments for the author, including concerns about dual publication, research ethics, or publication ethics. (Please upload your review as an attachment if it exceeds 20,000 characters)

Reviewer #1:

This study makes a highly valuable contribution to wildlife conservation genomics by establishing a curated mitochondrial genome reference database for China's state key protected wild mammals. One of its most commendable aspects is the integration of newly sequenced mitogenomes, especially from historical museum specimens, into a national-scale reference framework. This approach not only addresses critical taxonomic gaps but also enhances the practical utility of molecular tools in forensic identification and legal enforcement.

**Response: We are very grateful to the reviewer for their positive and encouraging comments on our manuscript. We are pleased that they found our study to be a valuable contribution and recognized the importance of integrating historical museum specimens into a national-scale reference framework for enhancing forensic applications.**

The authors are encouraged to further expand this database in future work by including population-level variation to support more nuanced applications such as local provenance inference. While the study successfully curated mitogenomes for 164 mammalian species, most of the newly sequenced data were obtained from single individuals or specimens from a limited number of localities (e.g., museum skins, feces, or archived samples). This narrow sampling does not capture the potential intraspecific variation across different geographic regions within China, which is especially relevant for widespread or genetically structured species. If possible, authors should include a statement acknowledging the current limitation in geographic coverage and propose plans for future work that incorporate population-level sampling across the full distribution ranges of species. I just have some minor suggestions for this important work.

**Response: We thank the reviewer for this important point. We agree completely that capturing intraspecific variation is critical for the forensic application of this database, especially for widespread species. We have acknowledged this limitation and highlighted** **the need for broader geographic sampling as a key future direction in our revised discussion.**

**We direct the reviewer to the third paragraph of the section "Application, future directions, and proposed best practices" which states:** *"Second, our results demonstrate that reliance on single or geographically narrow reference sequences can be misleading for widespread species, in which intraspecific genetic structure can be substantial, and capturing this variation is critical. Incorporating mitogenomes from across the geographic ranges of widespread species will enable the assignment of confiscated specimens to specific populations or origins. Such resolution is vital for tracking illegal wildlife trade, as demonstrated in recent pangolin studies [49]..."*

While the manuscript is generally well written and scientifically sound, here are a few grammar and phrasing issues identified in the abstract and introduction.

Others:

1. changed "...resulting in a refined list of 169 mammalian species." To "...resulting in a refined list of 169 mammalian species that are protected."

**Response: We thank the reviewer for this suggestion. We revised it** **(lines 35 and 277).**

2. Change "To capture nation-specific conservation need..." to "To capture nation-specific conservation needs..."

**Response: We thank the reviewer for this suggestion. We revised it (lines 55).**

Reviewer #2:

The manuscript presents a valuable and timely resource—a curated mitochondrial genome reference database for state-protected wild mammals in China. This dataset will support wildlife forensic applications, conservation genetics, and biodiversity monitoring. The combination of newly generated mitogenomes, historical museum specimens, and curated public databases demonstrates an integrated and innovative approach. The integration of updated taxonomic frameworks with genomic resources addresses a well-recognized gap in wildlife conservation and law enforcement, providing a reference standard for species identification. The successful generation of mitogenomes from archived museum specimens highlights the enduring value of natural history collections for genomic research.

**Response: We are deeply grateful to the reviewer for their thorough and highly positive evaluation of our manuscript. We are particularly encouraged that they recognized the value of our work, as well as the innovative nature of our integrated approach.**

However, there are some issues to be resolved:
Major:
1. The updated species list (169 taxa) is based on taxonomic evidence; however, it would strengthen the paper if the decision-making framework (criteria for synonymization, elevation, or exclusion) were described in more detail.

**Response: This is a great question. To address this, we have revised the “Materials and methods” section (under the subheading “Updating the List of State Key Protected Wild Mammals in China”).**

**The revised section now reads: “***To update the taxonomy of species in the 2021 LSKPWA, we established a decision-making framework that integrates the latest expert consensus from key national publications [5, 7] and the internationally recognized Mammal Diversity Database v2.3 (https://zenodo.org/records/17033774; last update September 2, 2025), which is actively curated by the American Society of Mammalogists.* *Each revision was* *cross-referenced with the latest peer-reviewed publications and verified by relevant experts.*”

2. The rationale for focusing solely on mitochondrial genomes (as opposed to including nuclear markers) is clear for forensic purposes, but could the authors briefly discuss limitations of mitogenomes for species delimitation, particularly in cases of introgression or hybridization?

**Response:** **We thank the reviewer for this suggestion. A discussion of the** **inherent limitations of mitogenomic data is indeed crucial for a balanced perspective. We have addressed this directly in our revised discussion by noting the challenges posed by unresolved taxonomy and natural hybridization, and by acknowledging that mitogenomes alone are not a panacea.**

**We have addressed this in the** **last two** **paragraphs of the revised discussion:** *"Fourth, our findings emphasize that the strength of forensic identification depends not only on genetic reference data but also on the robustness of the underlying taxonomy... Moreover, in some groups, natural hybridization may occur, further complicating identification and requiring more nuanced frameworks for forensic reporting.****"* And:** *"Finally, we acknowledge that mitochondrial genomes alone cannot fully resolve all cases of taxonomic or forensic ambiguity. Integration of nuclear and mitochondrial data... will provide the most robust framework for both taxonomy and conservation enforcement****."***

3. The paper mentions taxonomic issues in Moschus and Naemorhedus but does not provide explicit recommendations for resolving these ambiguities (e.g., need for additional sampling or integration of nuclear genomes). Could the authors clarify how these findings should inform future taxonomic revisions?

**Response: We appreciate the reviewer's request for more explicit recommendations. Our manuscript now clearly articulates that resolving these taxonomic issues is a prerequisite for the reliable forensic application of genetic data. We recommend a multi-faceted approach to address these challenges.**

**Our recommendations are stated in the** **last two paragraphs of the revised discussion:** *"...highlighting the need for systematic taxonomic revision as a prerequisite for effective application of genetic tools."* **And:** *"Finally, we acknowledge that mitochondrial genomes alone cannot fully resolve all cases of taxonomic or forensic ambiguity. Integration of nuclear and mitochondrial data, coupled with machine-learning approaches for species delimitation [48], which together will provide the most robust framework for both taxonomy and conservation enforcement."* **This explicitly recommends an** **integrative approach that includes nuclear genomics and advanced analytical methods to resolve the ambiguities in complex genera like *Moschus* and *Naemorhedus*.**

4. In terms of data availability and usability, the curated dataset is said to support forensic applications. Are the curated sequences provided in a searchable database format (e.g., BLAST-enabled)? If not, are there plans to implement such an interface to improve usability?

**Response: This is an excellent point regarding the practical usability of our curated data. While the current study focuses on establishing the curated dataset and proposing a new methodological standard for its use, we agree that developing a user-friendly interface will be a critical next step. Importantly, we recognize that simple BLAST searches (although it is pretty easy to achieve using just a few lines of codes using blast makeblastdb) and identifications** **based solely on genetic distance are not always reliable,** **particularly for taxa with low levels of intraspecific divergence. A phylogenetic framework** **should therefore be adopted to ensure more** **accurate** **species identification. In line with this view, we are in the process of formulating a new technical standard for wildlife forensic identification, which we will soon submit to the relevant authorities for review.**

Minor:
1. Line 163: The author provides the abbreviation of bootstrap value (BS), but BS should correspond to bootstrap rather than bootstrap value. However, the full name of a single word should be provided.

**Response: We thank the reviewer for this suggestion. We used “****bootstrap support (BS)” in this version.**

2. Figures 1 and 2 are informative, but some node labels and bootstrap values are difficult to read at the provided resolution. Consider enhancing the figure clarity or providing vector formats in the supporting information.

**Response: We thank the reviewer for this suggestion. In this version,** **nodes with BS** **≥ 95 are indicated by** **black dots, while those with BS below 95 are shown as numbers. Adjacent species in figures are** **separated by the same color with different transparency.**

3. The term “Maximum-likelihood” appears in both legends of Figures 1 and 2, and is abbreviated as “ML” in the legend of Figure 2. Normally, the abbreviation should be given when the phrase appears for the first time.

**Response: We thank the reviewer for this suggestion.** **We revised it.**

4. Please use the recommended citation format of this journal.

**Response: We thank the reviewer for this suggestion. We** **revised it.**
